# Breaking satellite silence: human satellite II RNA expression in ovarian cancer

**DOI:** 10.1172/JCI161981

**Published:** 2022-08-15

**Authors:** Shridar Ganesan

**Affiliations:** Department of Medicine and Pharmacology, Rutgers Robert Wood Johnson Medical School, and Rutgers Cancer Institute of New Jersey, Rutgers, The State University of New Jersey, New Brunswick, New Jersey, USA.

## Abstract

Multiple cancer types demonstrate abnormal expression of repetitive RNA sequences as a form of epigenetic instability. There is growing interest in understanding the role of repetitive RNAs in cancer pathogenesis and immunogenicity and in their potential role as diagnostic or therapeutic biomarkers. In this issue of the *JCI*, Porter and colleagues report on satellite RNA in a subset of ovarian cancers. The authors found that high expression of human satellite (HSAT) repeats — but not other families of repeats — was associated with an immunosuppressive phenotype in ovarian cancer cell lines and tumor samples. Further induction of HSAT RNA levels in vitro, surprisingly, leads to innate immune activation, suggesting a potential therapeutic strategy. This work highlights the expanding role of repetitive RNAs in tumor biology and the need to better define specific classes of repetitive elements expressed in cancer — as well as their role in tumorigenesis, tumor immunity, and the host response to cancer.

## Repetitive elements in the genome

Repetitive elements constitute a substantial proportion (more than 50%) of the human genome and include interspersed repeats and tandem repeats (1, 2). Interspersed repeats include the transposable elements (TE), such as short and long interspersed retrotransposable elements (SINE and LINE), and the long terminal repeat-containing human endogenous retroviruses (HERVs). Most transposable elements are stably silenced by DNA methylation and histone silencing marks. The tandem repeats include satellite repeats, microsatellite repeats and telomeric repeats, and are often involved in structural organization of chromosomes and found at or near centromeres and telomeres. α-Satellite repeats are organized in large arrays at all human centromeres. They can produce transcripts in normal cells that are localized in cis to centromeric regions and may be required for efficient loading of centromeric proteins and assembly of functional centromeres. Human satellite II (HSATII) is a satellite repeat family that is found in pericentromeric regions of a subset of human chromosomes, including chromosomes 1 and 16, which have especially large repeat regions. (5). These HSATII satellites are usually transcriptionally repressed and organized into constitutive heterochromatin.

## Repetitive RNAs in cancers

Abnormal expression of repetitive RNA, include TE and satellite repeats, have been reported in multiple cancers, often associated with global DNA demethylation, and may represent a form of epigenetic instability associated with tumorigenesis (5, 6). Repetitive RNA can form double-stranded RNA structures through internal repeats, which can be sensed in the cytoplasm by innate immune pathways evolved to detect viral RNA. These pathways include Toll-like receptors, RIG1, and other proteins, which, upon sensing dsRNA can activate interferon signaling (7, 8). Some transposable elements have intact open reading frames that can translate into an immunogenic protein. Abnormal expression of repetitive RNAs in cancers have been associated with evidence of innate immune activation and interferon signaling (9, 10). Pharmacologic approaches that increase the expression of repetitive elements, including DNA methylation inhibitors and histone modification inhibitors, have been proposed to activate local immune reactions in cancer, potentially increasing intrinsic cancer immunogenicity and enhancing responses to immune checkpoint inhibitors (11,12). There is growing interest in understanding the biological consequences of acquired epigenetic instability due to repetitive elements in cancer, both for diagnostic and therapeutic approaches.

## Satellite RNA in epithelial ovarian cancers

Porter and colleagues report in this issue of the *JCI* that a subset of epithelial ovarian cancers (EOCs) express high levels of satellite RNA, which was associated with an immunosuppressive phenotype (13). The investigators performed total RNA sequencing in a set of human cancer cell lines and identified a subset of ovarian cancer cell lines with high expression of satellite elements, including HSATII. High satellite RNA expression, even when present in cell lines that also expressed high levels of other repetitive elements families including HERVs, was positively correlated with gene signature of epithelial mesenchymal transition (EMT) and anticorrelated with interferon (IFN) response pathways. In contrast, cell lines that expressed HERVs and other TEs, but lacked HSATII expression, showed evidence of robust interferon pathway activation.

These findings suggest that HSATII and other satellite repeat expression may play a key role in modulating immune activation in cancer. The association of HSATII expression with EMT and its anticorrelation with IFN was confirmed in a series of EOC samples. Further, analysis of HSATII expression by RNA in situ hybridization in a small cohort of patients with EOCs demonstrated that high HSATII expression was associated with poor outcome. The investigators also showed that extracellular vesicles (EVs) derived from EOC cell lines contained high levels of HSATII RNA as well as other classes of repeat RNA, and suggested that these EVs may deliver HSATII RNAs to other cells, including immune cells, in the tumor microenvironment. Treatment of cell lines with DNA methylation inhibitors induced interferon signaling and expression of TE, including ERVs, LINEs, and SINEs, but not SAT elements. Conversely, treatment with histone-deacetylase inhibitors in multiple cell lines could induce SAT expression but did not strongly induce interferon signaling (13). These findings suggest that manipulating DNA methylation and histone acetylation may have differential effects on different families of repetitive elements. Transfection of locked-nucleic acids targeting HSATII into cell lines with high baseline HSATII expression, paradoxically, further increased HSATII RNA expression and triggered an IFN response, increased MHC1 protein expression, and decreased cell growth (13). This finding suggests that further induction of HSATII expression in HSATII-expressing tumors may break the immunosuppressive phenotype and induce immunogenicity. As Porter et al. have previously identified HSATII expression in pancreatic and other cancers, this finding may have relevance to multiple cancer types (14, 15).

## Conclusions

The observations by Porter and colleagues are provocative and demonstrate a complex relationship between repetitive HSATII expression and immunophenotype in ovarian cancer that will require further work to unravel (13). In particular, several important questions are raised. First, what is the mechanism of elevated SAT and HSATII RNA expression in ovarian cancer? The authors posit a role for *p53* mutation in the induction of SAT RNA expression (16). However almost all high-grade ovarian cancers have mutant TP53, but only a subset have HSATII expression, suggesting that there are other factors involved, or that only specific mutant alleles of TP53 are functional. The EOC genome is characterized by many structural variations and rearrangements, and the relationship between genomic instability seen in these cancers and the epigenetic instability related to repeat silencing is unclear. Does abnormal expression of SAT RNA disrupt centromere function and contribute to genomic instability, or does underlying genomic instability of these cancers disrupt normal chromatin architecture and contribute to abnormal expression of satellite RNA? In the case of *BRCA1,* loss of the gene induces structural genomic chaos due to defects in DNA repair but also induces derepression of satellite repeats and disruption of centromere function (17, 18). It would be informative to see if expression of HSATII in EOC and other cancers is associated with specific patterns of chromosomal rearrangements or specific defects in BRCA1-related DNA repair pathways.

It is also puzzling why the expression of satellite RNA, as opposed to that of TE RNA, leads to an immunosuppressive phenotype associated with EMT instead of triggering an IFN response. The nucleus of some cancer cell lines can retain abnormally expressed HSATII transcripts in large foci where they form complexes with MeCP2 and other chromatin regulatory factors, leading to reorganized distribution of polycomb-related protein in the nucleus (5). This sequestering of RNA in the nucleus may prevent RNA sensing systems from recognizing repeat RNAs in the cytoplasm. Similarly, the redistribution of key polycomb-associated genes could affect transcriptional programs and contribute to the EMT phenotype associated with HSATII expression.

The finding by Porter and colleagues highlights the growing importance of acquired epigenetic instability in cancers and demonstrates the complex biology associated with different classes of repetitive elements and the consequences of their dysregulation in cancer (13). Methods to more robustly identify, categorize, and measure expression of different classes of repetitive elements will be needed to further define their roles in tumorigenesis, tumor immunity, and the host response to cancer.

## Version 1 08/15/2022

Electronic publication
